# A comparative study of the substrate preference of the sialidases, CpNanI, HpNanH, and BbSia2 towards 2-Aminobenzamide-labeled 3′-Sialyllactose, 6′-Sialyllactose, and Sialyllacto-N-tetraose-b

**DOI:** 10.1016/j.bbrep.2024.101791

**Published:** 2024-07-19

**Authors:** Madhu Lata, T.N.C. Ramya

**Affiliations:** a CSIR- Institute of Microbial Technology, Sector 39-A, Chandigarh, 160036, India; b Academy of Scientific & Innovative Research (AcSIR), Ghaziabad, Uttar Pradesh, 201002, India

**Keywords:** Sialidase, HpNanH, CpNanI, BbSia2, LSTb, DSLNT, Sialyllactose, Glycosidic linkage, Siaα2-6GlcNAc

## Abstract

Sialidases catalyze the removal of terminal sialic acids from sialylated biomolecules, and their substrate preference is frequently indicated in terms of the glycosidic linkages cleaved (α2-3, α2-6, and α2-8) without mention of the remaining sub-terminal reducing-end saccharide moieties. Many human gut commensal and pathogenic bacteria secrete sialidases to forage for sialic acids, which are then utilized as an energy source or assimilated into membrane/capsular structural components. Infant gut commensals similarly utilize sialylated human milk oligosaccharides containing different glycosidic linkages. Here, we have studied the preference of the bacterial sialidases, BbSia2 from *Bifidobacterium bifidum*, CpNanI from *Clostridium perfringens*, and HpNanH from *Glaesserella parasuis,* for the glycosidic linkages, Siaα2-3Gal, Siaα2-6Gal, and Siaα2-6GlcNAc, by employing 2-Aminobenzamide-labeled human milk oligosaccharides, 3′-Sialyllactose (3′-SL), 6′-Sialyllactose (6′-SL), and Sialyllacto-N-tetraose-b (LSTb), respectively, as proxies for these glycosidic linkages. BbSia2, *Cp*NanI, and HpNanH hydrolyzed these three oligosaccharides with the glycosidic linkage preferences, 3**′**-SL (Siaα2-3Gal) ≥ LSTb (Siaα2-6GlcNAc) ≥ 6**′**-SL (Siaα2-6Gal), 3**′**-SL (Siaα2-3Gal) ≥ 6**′**-SL (Siaα2-6Gal) > LSTb (Siaα2-6GlcNAc), and 3′-SL (Siaα2-3Gal) ≥ 6′-SL (Siaα2-6Gal) > LSTb (Siaα2-6GlcNAc), respectively. Our finding suggests that sub-terminal reducing-end saccharide moieties can profoundly influence the substrate preference of sialidases, and advocates for the characterization and indication of the substrate preference of sialidases in terms of both the glycosidic linkage and the sub-terminal reducing-end saccharide moiety.

## Highlights

• Sialidases can act on Siaα2-3Gal, Siaα2-6Gal and Siaα2-6GlcNAc linkages.
• BbSia2 from *Bifidobacterium bifidum* prefers 3**′**-SL ≥ LSTb ≥ 6**′**-SL.
• CpNanI from *Clostridium perfringens* prefers 3**′**-SL ≥ 6**′**-SL > LSTb.
• HpNanH from *Glaesserella parasuis* prefers 3′-SL ≥ 6′-SL > LSTb.

## 1 Introduction

Sialidases are hydrolytic enzymes that cleave terminal sialic acids from sialylated molecules, and they fall in the GH33 (bacterial sialidases, *trans*-sialidases, and human sialidase), GH156 (bacterial exo-sialidases), GH34 (viral sialidases), GH58 (viral sialidases), and GH83 (viral sialidases) families of the CAZy database [1]. Sialidases represent a key weapon in the arsenal employed by human commensal and pathogenic microbes to survive in the sialic acid-rich glycan landscape of their ecological niche [2,3]. This is because the released sialic acid is utilized as an energy source, for instance, by *nanLET* operon enzymes, *nanL*: aldolase, *nanE*: manNAc/N-acetylglucosamine epimerase, and *nanT*: transporter facilitator in *Bacteroides fragilis* [4], and/or assimilated into lipooligosaccharide/capsular structures by sialic acid assimilating enzymes such as α2-3-sialyltransferase (*lsgB* product) acting in concert with NeuA (sialic acid O-acetylesterase), SiaB (cytidine monophosphate-sialic acid synthetase) and NanH (sialidase) [5,6] in *Glaesserella* (formerly *Haemophilus*) *parasuis* [7], *Glaesserella influenzae* [8], *Clostridium perfringens,* and *Escherichia coli* [9,10].

Human milk oligosaccharides (HMOs) are a diverse group of prebiotic oligosaccharides [11] that promote the selective growth and colonization of beneficial microbes such as bifidobacteria and lactobacilli [[12], [13], [14]], and represent an abundant source of sialic acid in the infant gut. HMOs represent the third largest component (9–22 g/L) of human milk and include sialylated HMOs that contain the glycosidic linkages, Siaα2-3Gal, Siaα2-6Gal, and/or Siaα2-6GlcNAc [15]. Sialylated HMOs include 3′-Sialyllactose (3′-SL), 6′-Sialyllactose (6′-SL), Sialyllacto-N-tetraose-a (LST a), Sialyllacto-N-tetraose-b (LSTb), Sialyllacto-N-tetraose-c (LSTc), Disialyllacto-N-tetraose (DSLNT), Sialylfucosyllacto-*N*-tetraose (F-LSTa), Fucosylsialyllacto-*N*-tetraose (F-LSTb), Fucosylsialyllacto-N-hexaose (FS-LNH), Fucosylsialyllacto-N-neohexaose I (FS-LNnH I), and Fucosyldisialyllacto-N-hexaose II (FDS-LNH II) [15], and are important for the developing brain in infants as sialylated gangliosides and glycoproteins are involved in increasing synaptogenesis in the infant's developing brain cortex [16]. Commensal microbes in the infant's gut metabolize the HMOs by the enzymatic release of monosaccharides from the oligosaccharide structure, with terminal monosaccharides like fucose and sialic acid usually being cleaved off first [17,18].

**Fig. 1:**
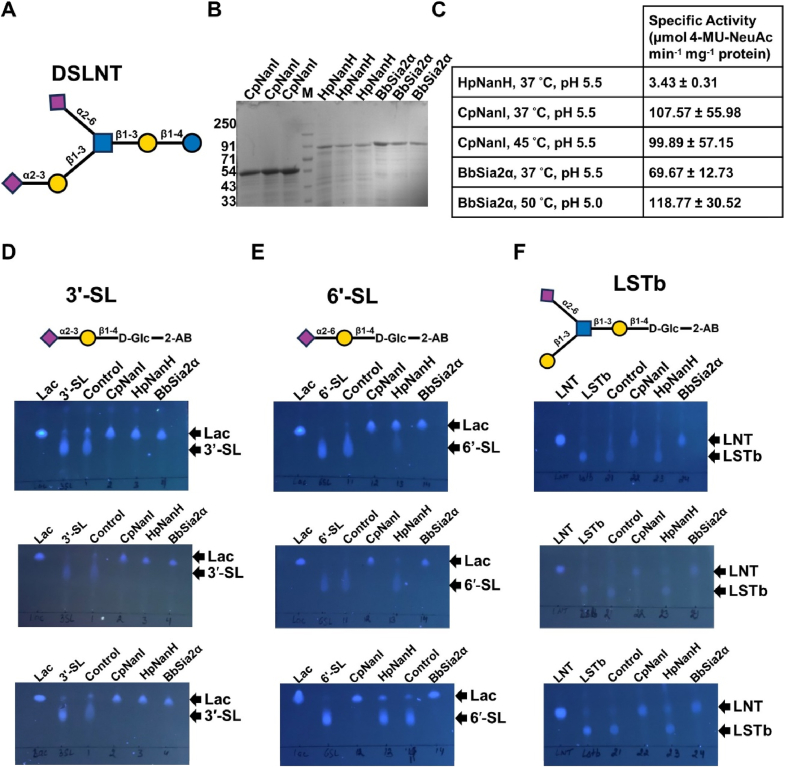
**Sialidase activity of BbSia2, CpNanI, and HpNanH, on the 2-AB-labeled HMOs, 3′-SL, 6′-SL, and LSTb.** (A) The SNFG representation of DSLNT. (B) The Coomassie-stained SDS-PAGE shows the three independent preparations of purified proteins, CpNanI, HpNanH, and BbSia2α used in the biochemical assays. M: Molecular weight marker from PureGene. (C) Specific Activity of HpNanH, CpNanI, and BbSia2α for the synthetic substrate, 4-MU-Neu5Ac, at the specified reaction conditions. (D**, E, F**) UV-fluorescence of TLC plates showing the sialidase activity of CpNanI (1 μM), HpNanH (1 μM), and BbSia2α (1 μM) on the 2-AB-labeled sialylated oligosaccharides, **(D)** 3′-SL (2.9 mM), **(E)** 6′-SL (2.7 mM) and **(F)** LSTb (2.9 mM) following a reaction time of 24 h. “Control” refers to the reaction mix minus sialidase. The TLC solvent used was Butanol: Acetic Acid: Water :: 2:1:1. Three biological replicates for each substrate are shown. The structures of the 2-AB-labeled oligosaccharides, 3′-SL, 6′-SL, and LSTb are represented above the TLC images for reference.

Several sialidases, including those of commensal and pathogenic gut microbes, have been biochemically characterized for their substrate preference [19]. Except for a few such as *Macrobdella decora* (SL) [2] that have exclusive specificity towards Siaα2-3Gal linkage, most GH33 sialidases harbour the ability to hydrolyze sialic acids in α-2,3, α-2,6, and α-2,8 linkages, with varying orders of preference [19]. For instance, both *C. perfringens* sialidase (CpNanI) and *B. bifidum* sialidase (BbSia2) prefer Siaα2-3Gal over Siaα2-6Gal; CpNanI also cleaves Siaα2-8Sia with the substrate preference order Siaα2-3Gal > Siaα2-6Gal > Siaα2-8Sia [20], and BbSia2 also likely cleaves the Siaα2-6GlcNac linkage as Lacto-N-tetraose (LNT) was also observed as a product in addition to monosialyl-LNT upon incubation with DSLNT [18], which has this linkage (Fig. 1A). *Bifidobacterium longum* sialidases (NanH1 and NanH2) display the substrate preference order, Siaα2-6Gal > Siaα2-3Gal, and *Clostridium tertium* sialidase (NanH) and *B. fragilis* sialidase (BfGH33A, BfGH33B, and BfGH33C) display the substrate preference, Siaα2-8Gal > Siaα2-3Gal > Siaα2-6Sia, and Siaα2-8Gal > Siaα2-3Gal ∼ Siaα2-6Sia, respectively. Interestingly, despite the body of literature about the substrate preference of sialidases, with the exception of BbSia2, the ability of sialidases to hydrolyze substrates, such as DSLNT, with Siaα2-6GlcNAc has not been studied.

Here, we have studied the substrate preference of the bacterial sialidases, *Bb*Sia2 from *B. bifidum*, *Cp*NanI from *C. perfringens*, and *Hp*NanH from *G. parasuis,* for the glycosidic linkages, Siaα2-3Gal, Siaα2-6Gal, and/or Siaα2-6GlcNAc, by employing the 2-Aminobenzamide-labeled human milk oligosaccharides, 3′-SL, 6′-SL, and LSTb, respectively, as proxies for these glycosidic linkages. We show that the sub-terminal reducing-end saccharide moiety can markedly influence the substrate preference of sialidases.

## 2 Material and methods

### 2.1 Expression, purification, and specific activity determination of recombinant CpNanI, HpNanH and BbSia2

The genes encoding the proteins, CpNanI (NCBI Accession number WP_011590331.1, amino acids 243 to 694, corresponding to the protein construct used for structural characterization [21], HpNanH (NCBI Accession number WP_051502694.1, amino acids 29 to 803), and BbSia2 (Genbank Accession number AB278567.1, amino acids 36 to 768; this construct lacks the C-terminal unstructured region and is referred to as BbSia2α henceforth) were cloned in the pET28a(+) vector in the *Nco*I and *Xho*I sites to encode a hexahistidine tag at the C-terminus, and the recombinant clones were transformed into *Escherichia coli* BL21(DE3) cells. For protein expression, an overnight-grown primary culture of the transformed BL21 cells was used to inoculate Luria Bertani medium containing Kanamycin (30 μg/ml). The culture was incubated at 37 °C with continuous shaking up to an OD_600_ of 0.6–0.8. The protein expression was induced with 1 mM Isopropyl β-**D**-1-thiogalactopyranoside (IPTG) (incubated at 37 °C for 4 h with 200 rpm), 0.1 mM IPTG (incubated at 22 °C for 10 h with 180 rpm), and 0.1 mM IPTG (incubated at 37 °C for 4 h with 200 rpm), respectively, for CpNanI, HpNanH, and BbSia2α. The cells were harvested by centrifugation at 4000×*g* for 7 min. The C-terminal hexa-histidine tagged recombinant proteins were purified using Ni-NTA affinity chromatography. The cells were resuspended in lysis buffer (20 mM Tris, 150 mM NaCl pH 7.5, and 0.5% N-lauryl sarcosine) and disrupted using an ultrasonicator for 30 min (amplitude 25, 10-10 s pulse on and off). The cell lysate was centrifuged at 11000 rpm for 30 min. The cleared lysate was incubated with Ni-NTA affinity resin at 4 °C with end-over-end rotation for 2 h on a rotary mixer. The beads were washed with wash buffer (20 mM Tris, 150 mM NaCl pH 7.5, 25 mM imidazole) and the proteins were eluted using elution buffer (20 mM Tris, 150 mM NaCl pH 7.5, 250 mM imidazole). The protein purity was assessed by sodium dodecyl sulfate-polyacrylamide gel electrophoresis (SDS-PAGE), pure fractions were pooled together and dialyzed extensively against Tris-buffered saline (20 mM Tris, 150 mM NaCl pH 7.5). The dialyzed proteins CpNanI (51621.48 Da), HpNanH (88088.64 Da), and BbSia2α (77807.82 Da) (biological triplicates of each protein) were analyzed by SDS-PAGE (Fig. 1B) and the concentration of protein was estimated at OD_280_ (DS-11, DeNovix and Nanodrop, ThermoScientific spectrophotometers). Protein concentrations were also verified with a Bradford assay reagent (Sigma), and found to be within the same range. The specific activity of the proteins was estimated by measuring the activity of the proteins with the synthetic substrate 4-Methylumbelliferyl-α-D-N-acetylneuraminic acid (4-MU-Neu5Ac) and using the OD_280_ measurement-derived protein concentrations. The sialidase activity was measured using 160 μM 4-MU-Neu5Ac in 50 mM sodium acetate, pH 5.5 at 37 °C for all proteins, and additionally using 160 μM 4-MU-Neu5Ac in 50 mM sodium acetate, pH 5.5 at 45 °C for CpNanI and 50 mM sodium acetate, pH 5.0 at 50 °C for BbSia2α. The amount of product formed (4-MU) was measured (at a gain of 60) every minute for an hour using a Biotek Cytation 5 Multimode plate reader. Reaction rates were calculated by using the initial linear portion of the progress curve and converting the fluorescence measured to 4-MU concentration using a standard plot of 4-MU fluorescence versus concentration. The optimal temperature and pH for the sialidase reaction were also similarly determined using 5 μM (BbSia2α) or 10 μM (CpNanI and HpNanH) 4-MU-Neu5Ac and 0.01 nM (CpNanI) or 0.01 μM (HpNanH) or 0.25 nM (BbSia2α) protein. For optimizing temperature, 50 mM sodium acetate, pH 5.5 was used. For optimizing pH, 45 °C (CpNanI) and 37 °C (HpNanH) were used. The following buffers were used for the different pH conditions - 100 mM Glycine-HCl (pH 2.5), 50 mM sodium acetate (pH 4.5 or pH 5.5), 20 mM Tris(hydroxymethyl)aminomethane in 150 mM sodium chloride (pH 7.5), and 100 mM Glycine-NaOH (pH 9.2).

### 2.2 Preparation of labeled oligosaccharides for the sialidase assays

3′-SL (16617) and 6′-SL (17866) were from Cayman Chemicals, and LSTb (00–018) was from Glycotech. 3′-SL, 6′-SL, and LSTb were enriched (away from non-sialylated impurities) using AG1X-8 Dowex resin (kind gift from Prof. Surolia), labeled with 2-Aminobenzamide (2-AB), and purified by HILIC-SPE.

***Oligosaccharide purification using AG1X-8 Dowex resin****:* AG1X-8 Dowex resin, 500 mg, was conditioned with 3 M ammonium formate and washed 10 times with water. The oligosaccharide sample was loaded on a column, washed, and eluted sequentially with water, 0.01 M formic acid, 1 M formic acid, 4 M formic acid, and 0.4 M ammonium formate in 4 M formic acid. Each eluate fraction was vacuum concentrated to evaporate the solvent, resuspended in 30 μL of water, and analyzed by *N,N*-Diphenylamine staining for the presence of sialylated oligosaccharides following thin-layer chromatography (TLC) on Silica gel 60 F_254_ (Merck Millipore) sheets (Fig. S1). Fractions with sialylated oligosaccharides were pooled together in water for labeling.

***Labeling of oligosaccharides with Anthranilamide (*2-AB*) and Purification using HILIC Column:*** Oligosaccharides were labeled with Anthranilamide (2-AB) using 2-Picoline borane (2-Methylpyridine borane complex) as the reducing agent [22]. The freshly prepared solutions of 2-AB (48 mg/ml in dimethyl sulfoxide (DMSO) containing 15% Glacial acetic acid) and reducing agent 2-Picoline borane (1 M in DMSO) were mixed with oligosaccharide in 1:1:2 ratio. The mixture was vortexed for 5 min and was incubated at 65 °C for 2 h [22], and subsequently purified by hydrophilic interaction liquid chromatography (HILIC) solid phase extraction (SPE) [23,24]. Briefly, the pooled purified fractions (20 μL) of the sialylated oligosaccharide sample reconstituted in 480 μL of acetonitrile was loaded onto a hand-packed SPE column comprising a 1 ml syringe with 50 mg cotton (washed with 100% acetonitrile and pre-conditioned with 96% acetonitrile). The column was washed with 96% acetonitrile and eluted with water. The eluates were vacuum concentrated to evaporate solvent and finally were resuspended in 30 μL of water. A 0.5 μL volume of each fraction was analyzed by TLC for the presence of 365 nm UV-active spots (Fig. S1), and the fractions with labeled oligosaccharides pooled together. The oligosaccharide concentration was estimated by spectrophotometry using the extinction coefficient reported for 2-AB (CID 6942) at 335 nm in PUBCHEM [25].

**Sialidase assays of CpNanI, HpNanH, and BbSia2α on the sialylated substrates 3′-SL, 6′-SL, and LSTb:** For preliminary assessment of the ability of the sialidases to accept 3′-SL, 6′-SL, and LSTb as substrates, we used the sialidases at a final concentration of 1 μM and 2-AB labeled oligosaccharides (at final concentrations 2.9 mM of 3′-SL, 2.7 mM of 6′-SL and 2.9 mM of LSTb) in 50 mM sodium acetate, pH 5.5, and incubated the reaction mix for 24 h at 37 °C. For further assessing the substrate preference, ten-fold serial dilutions of the sialidases (with final concentrations in the range 1 μM to 0.1 nM) were mixed with 2-AB labeled sialylated oligosaccharide substrates (to a final concentration of 3.2 mM) in 50 mM sodium acetate buffer, pH 5.5 (for CpNanI and HpNanH) and pH 5.0 (for BbSia2α). The reaction mixes were incubated for an hour at static temperature conditions of 45 °C, 37 °C, and 50 °C for CpNanI, HpNanH, and BbSia2α, respectively. After 1 h, reactions were stopped by heat denaturation of the enzymes in the reaction mixture (95 °C for 10 min). A 0.5 μL volume of each reaction mixture was analyzed by TLC on a silica gel 60 F_254_ sheet, labeled oligosaccharides were visualized under a UV lamp at 365 nm wavelength, and the image was captured using the camera on an iPhone10R. The intensities of the substrate and product spots visualized by TLC were measured using public domain NIH ImageJ 1.54g software (developed at the U.S. National Institutes of Health and available on the Internet at http://rsb.info.nih.gov/nih-image/) and the “mean” intensity values generated by ImageJ were used to calculate the fraction of substrate converted to product at different enzyme concentrations.

## 3 Results

We made three independent preparations of the recombinant sialidases, CpNanI, HpNanH, and BbSia2α (Fig. 1B), and verified sialidase activity using the synthetic substrate, 4-MU-Neu5Ac (Fig. 1C). All the protein preparations were enzymatically active with reasonable variation in specific activity between the three preparations (Fig. 1C). HpNanH displayed the least specific activity for 4-MU-Neu5Ac, which was ∼30-fold and ∼20-35-fold lower than that of CpNanI and BbSia2α, respectively, considering the same reaction conditions for all enzymes (sodium acetate buffer, pH 5.5, 37 °C) as well as the different, individually optimized reaction conditions - sodium acetate buffer, pH 5.5, 37 °C for HpNanH (Fig. S2), sodium acetate buffer, pH 5.5, 45 °C for CpNanI (Fig. S2), and sodium acetate buffer, pH 5.0 [18], 50 °C (Fig. S2) for BbSia2α. CpNanI and BbSia2α completely liberated sialic acid from the oligosaccharides, 3**′**-SL, 6′-SL, and LSTb, following a reaction time of 24 h in sodium acetate, pH 5.5 (Fig. 1D–F). In contrast, under the same conditions, HpNanH catalyzed complete hydrolysis of 3**′**-SL and partial/minimal hydrolysis of 6**′**-SL and LSTb in 24 h (Fig. 1D–F).

**Fig. 2:**
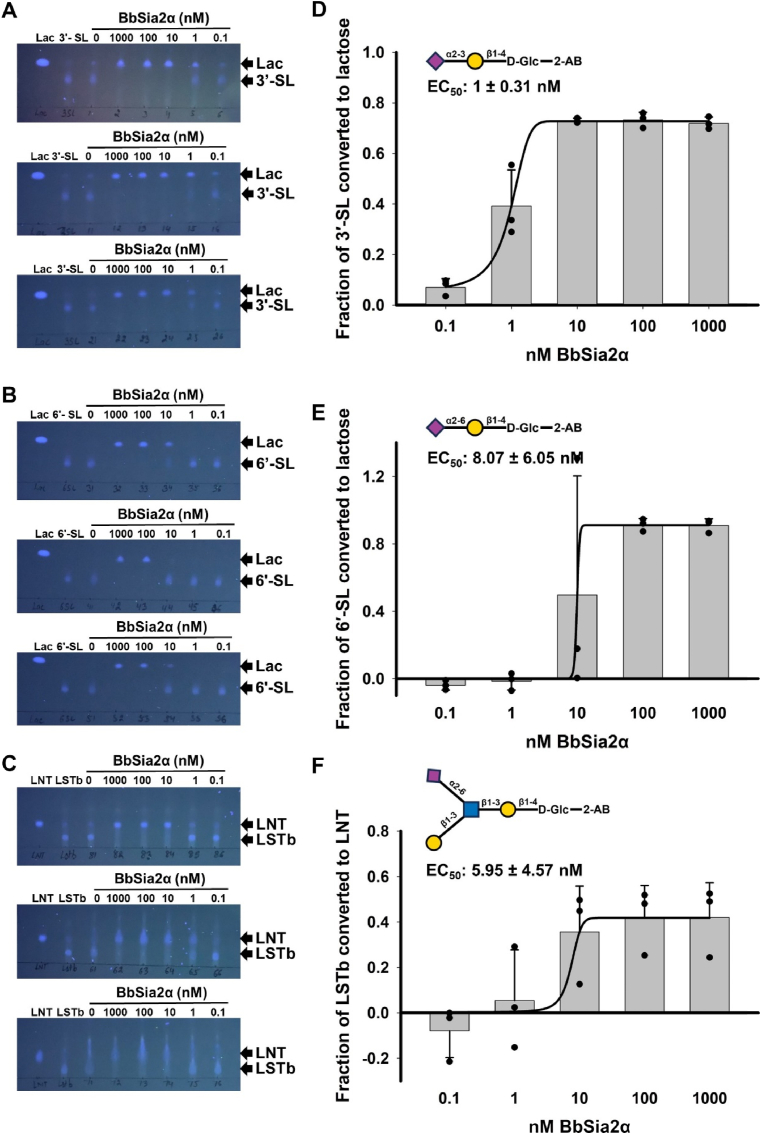
**Substrate preference of the sialidase BbSia2 towards** the 2-AB-labeled HMOs, 3′-SL, 6′-SL, and LSTb**. (A, B, C)** UV-fluorescence of TLC plates showing the hydrolysis of 3.2 mM 2-AB-labeled sialylated oligosaccharides, 3′-SL (A), 6′-SL **(B)**, and LSTb **(C)** by the sialidase BbSia2α (ten-fold serial dilutions starting from 1 μM final concentration in the reaction mix) (different concentrations of enzymes) following a reaction time of 1 h. Three biological replicates are shown here. The TLC solvent used was Butanol: Acetic Acid: Water :: 2:1:1.5. **(D, E, F)** The preference of the sialidase BbSia2α for the 2-AB-labeled sialylated HMO substrates, **(D)** 3′-SL, **(E)** 6′-SL, and **(F)** LSTb, represented by the fraction of substrate converted to the product with varying sialidase concentration. The results are plotted as means and the error bars represent standard deviations of the three biological replicates shown in A**-C** (three independent recombinant protein preparations). The structures of the 2-AB-labeled oligosaccharides, 3′-SL, 6′-SL, and LSTb are represented above the plots for reference.

To further explore the substrate preference of the sialidases, we analyzed the hydrolysis of 3**′**-SL, 6′-SL, and LSTb after just 1 h, using serial dilutions of the enzymes. Using three independent protein preparations as biological replicates, we found that BbSia2α, at a concentration of 10 nM or higher in the reaction mix, could completely hydrolyze 3**′**-SL (Fig. 2A). BbSia2α, at concentrations of 10 nM or 100 nM, resulted in partial to complete hydrolysis of LSTb and 6**′**-SL in the three biological replicates (Fig. 2B-C). However, we noted the appearance of product formation from 3**′**-SL and LSTb, but not 6**′**-SL at lower concentrations of BbSia2α (Fig. 2A-C). We calculated the EC_50_ values of BbSia2α for 3**′**-SL, 6**′**-SL, and LSTb to be 1 ± 0.31 nM, 8.07 ± 6.05 nM, and 5.95 ± 4.57 nM, respectively, with no statistically significant differences among these values (p-value>0.1, two-sample equal variance *t*-test) (Fig. 2D-F). Based on the sialidase activity at lower concentrations as well as on the (statistical significance of the) EC_50_ values calculated, we state the substrate preference of BbSia2α as 3**′**-SL (Siaα2-3Gal) ≥ LSTb (Siaα2-6GlcNAc) ≥ 6**′**-SL (Siaα2-6Gal) (Fig. 2A-Ff).

**Fig. 3:**
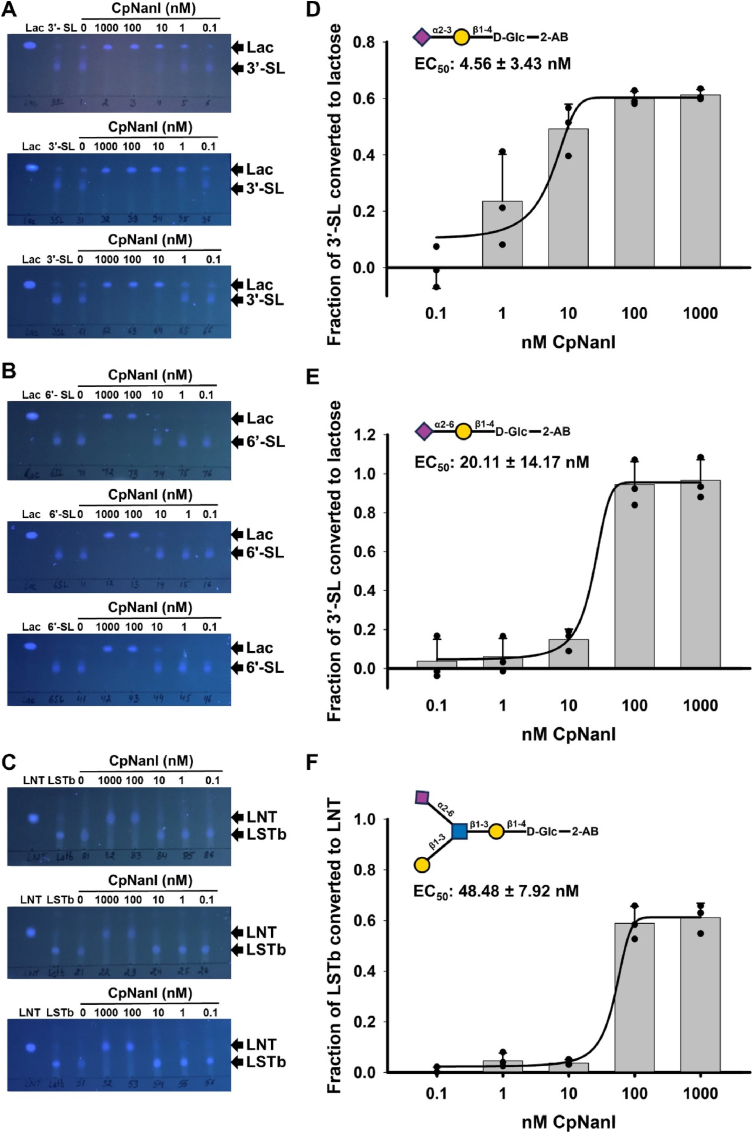
**Substrate preference of the sialidase CpNanI towards the** 2-AB-labeled **HMOs**, 3′-SL, 6′-SL, and LSTb**. (A, B, C)** UV-fluorescence of TLC plates showing the hydrolysis of 3.2 mM 2-AB-labeled sialylated oligosaccharides, 3′-SL (A), 6′-SL **(B)**, and LSTb **(C)** by the sialidase CpNanI (ten-fold serial dilutions starting from 1 μM final concentration in the reaction mix) (different concentrations of enzymes) following a reaction time of 1 h. Three biological replicates are shown here. The TLC solvent used was Butanol: Acetic Acid: Water :: 2:1:1.5. **(D,** E**,** F**)** The preference of the sialidase CpNanI for the 2-AB-labeled sialylated HMO substrates, **(D)** 3′-SL, **(E)** 6′-SL, and **(F)** LSTb, represented by the fraction of substrate converted to the product with varying sialidase concentration. The results are plotted as means and the error bars represent standard deviations of the three biological replicates shown in **a-c** (three independent recombinant protein preparations). The structures of the 2-AB-labeled oligosaccharides, 3′-SL, 6′-SL, and LSTb are represented above the plots for reference.

Using three independent protein preparations, we found that CpNanI, at a concentration of 100 nM or higher in the reaction mix, completely liberated sialic acid from 3**′**-SL, 6**′**-SL, and LSTb (Fig. 3A–C). However, we noted the appearance of product formation from 3**′**-SL, but not LSTb or 6**′**-SL at lower concentrations of CpNanI (Fig. 3A–C). We calculated the EC_50_ values of CpNanI for 3**′**-SL, 6**′**-SL, and LSTb to be 4.56 ± 3.43 nM, 20.11 ± 14.17 nM, and 48.48 ± 7.92 nM, respectively, with a significantly higher EC_50_ for LSTb compared to 3**′**-SL (p-value: 0.0009, two-sample equal variance *t*-test) and 6**′**-SL (p-value: 0.039, two-sample equal variance *t*-test) but no statistically significant difference between the EC_50_ for 3**′**-SL and 6**′**-SL (p-value>0.1, two-sample equal variance *t*-test) (Fig. 3D-F). Based on the sialidase activity at lower concentrations as well as on the (statistical significance of the) EC_50_ values calculated, we state the substrate preference of CpNanI as 3**′**-SL (Siaα2-3Gal) ≥ 6**′**-SL (Siaα2-6Gal) > LSTb (Siaα2-6GlcNAc) (Fig. 3A–F).

**Fig. 4:**
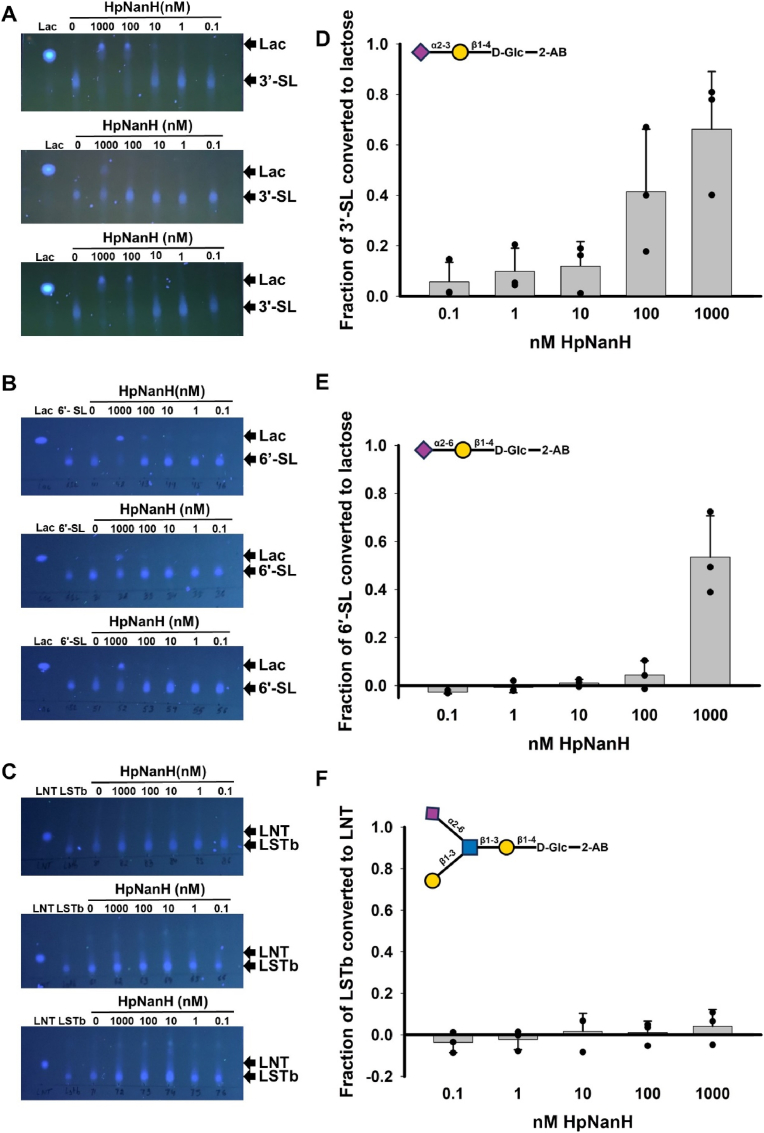
**Substrate preference of the sialidase HpNanH towards the 2-AB-labeled HMOs**, 3′-SL, 6′-SL, and LSTb**. (A, B, C)** UV-fluorescence of TLC plates showing the hydrolysis of 3.2 mM 2-AB-labeled sialylated oligosaccharides, 3′-SL (A), 6′-SL **(B)**, and LSTb **(C)** by the sialidase HpNanH (ten-fold serial dilutions starting from 1 μM final concentration in the reaction mix) (different concentrations of enzymes) following a reaction time of 1 h. Three biological replicates are shown here. The TLC solvent used was Butanol: Acetic Acid: Water :: 2:1:1.5. **(D, E, F)** The preference of the sialidase HpNanH for the 2-AB-labeled sialylated HMO substrates, **(D)** 3′-SL, **(E)** 6′-SL, and **(F)** LSTb, represented by the fraction of substrate converted to the product with varying sialidase concentration. The results are plotted as means and the error bars represent standard deviations of the three biological replicates shown in **a-c** (three independent recombinant protein preparations). The structures of the 2-AB-labeled oligosaccharides, 3′-SL, 6′-SL, and LSTb are represented above the plots for reference.

Using three independent protein preparations as biological replicates, we found that HpNanH, showed complete hydrolysis of 3**′**-SL at an enzyme concentration of 1000 nM and partial hydrolysis at an enzyme concentration of 100 nM in two of the biological replicates (Fig. 4A, D). In contrast, partial hydrolysis of 6**′**-SL and no hydrolysis of LSTb was observed when HpNanH was used at 1000 nM concentration under the same reaction conditions (Fig. 4B, C, E, F). Considering the absence of complete hydrolysis, we could not estimate EC_50_ of HpNanH for these substrates. Visual inspection indicated that HpNanH utilized 3′-SL and 6′-SL with the preference, 3′-SL (Siaα2-3Gal) ≥ 6′-SL (Siaα2-6Gal), and did not hydrolyze the Siaα-2,6GlcNAc glycosidic linkage in LSTb under these reaction conditions (Fig. 4A–F). Based on the sialidase activity observed and the minimal hydrolysis of LSTb in 24 h (Fig. 1F), we state the substrate preference of HpNanH as 3′-SL (Siaα2-3Gal) ≥ 6′-SL (Siaα2-6Gal) > LSTb (Siaα2-6GlcNAc).

## 4 Discussion

In the present study, we have explored the substrate preference of the bacterial sialidases, CpNanI, BbSia2α, and HpNanH for the glycosidic linkages, Siaα2-3Gal, Siaα2-6Gal, and Siaα2-6GlcNAc using 2-Aminobenzamide-labeled 3′-SL, 6′-SL, and LST-b as proxies for these glycosidic linkages. We have demonstrated that despite 3′-SL (Siaα2-3Gal) being the preferred substrate for all these sialidases, these sialidases differed widely in their preference for 6′-SL (Siaα2-6Gal) and LSTb (Siaα2-6GlcNAc). Whereas BbSia2α and CpNanI were able to hydrolyze Siaα2-6GlcNAc in LSTb in a reaction time of just 1 h, they did so with differing preferences - 3**′**-SL (Siaα2-3Gal) ≥ LSTb (Siaα2-6GlcNAc) ≥ 6**′**-SL (Siaα2-6Gal) and 3**′**-SL (Siaα2-3Gal) ≥ 6**′**-SL (Siaα2-6Gal) > LSTb (Siaα2-6GlcNAc), respectively. In contrast, HpNanH did not hydrolyze the Siaα2-6GlcNAc linkage in LSTb in a reaction time of 1 h, albeit minimal hydrolysis was observed at 24 h, and we predict the substrate preference to be 3′-SL (Siaα2-3Gal) ≥ 6′-SL (Siaα2-6Gal) > LSTb (Siaα2-6GlcNAc). HpNanH displayed lower specific activity for the synthetic substrate, 4-MU-Neu5Ac (Fig. 1c), and this could be the reason for the overall lower activity for the natural substrates, including 3**′**-SL.

In a previous study, Kiyohara et al. showed that the sialidase BbSia2α has a linkage preference for α2-3 sialic acid over α2-6 sialic acid, using 3′-SL, 6′-SL, and DSLNT as substrates [18]. Our present findings are in agreement and show a similar preference for α2-3 sialic acid-containing 3′-SL over α2-6 sialic acid-containing 6′-SL or LSTb for the sialidases, BbSia2α, CpNanI, and HpNanH. However, whereas the study by Kiyohara et al. also suggested that BbSia2α could hydrolyze the Siaα2-6GlcNAc linkage in DSLNT [18], it did not elaborate on the substrate preference for the different glycosidic linkages. To understand the linkage preference for α2-6 sialic acid, we have used two substrates, 6′-SL and LSTb, in this study, and we demonstrate that BbSia2α and CpNanI show distinct substrate preferences, with CpNanI preferring 6′-SL that contains Siaα2-6Gal linkage over LSTb that contains Siaα2-6GlcNAc linkage, in contrast to BbSia2α, while HpNanH only minimally hydrolyzed LST-b (Siaα2-6GlcNAc linkage) after 24 h. The contrasting substrate preferences of CpNanI and BbSia2α might stem from differences in the structural landscape of the active site, especially relating to the “+1 binding subsite”. An X-ray-diffracted crystal structure solution for Neu5Ac-complexed CpNanI is available (PDB ID: 2BF6) [26], and AlphaFold2-predicted structures are available for BbSia2α (AlphaFoldDB ID: A0A1Y1C8G4) and HpNanH (AlphaFoldDB ID: I7AJQ9); however, our attempts to understand differences in the substrate binding region by molecular docking studies of 3′-SL and 6′-SL with CpNanI were not successful as we were unable to obtain Neu5Ac positioned in the active site. It is hoped that this limitation will be addressed by future structural studies in this area.

It is pertinent to note other limitations of this study. One is that the substrate preferences reported here are with 2-Aminobenzamide-labeled HMOs, where the reducing-end monosaccharide (Glc) is in a linearized form as it is derivatized with 2-AB; future studies could conjugate the label after the reaction to assess the preference on intact HMOs. Another point to note is that whereas we have used the substrates, 3′-SL, 6′-SL, and LST-b as proxies for the glycosidic linkages, Siaα2-3Gal, Siaα2-6Gal, and Siaα2-6GlcNAc, other constituent moieties, such as the two Gal moieties in LST-b, which could be accommodated in subsites of the sialidase active site, might also contribute towards overall substrate preference.

Beyond structural differences in the active site, the enzyme activity of carbohydrate-active enzymes is frequently modulated by the presence of co-occurring non-catalytic Carbohydrate-Binding Modules (CBMs). CBMs are known to play important roles in the catalysis of insoluble polysaccharides via proximity, targeting, and disruptive effects [27], and might also enhance the catalytic activity for aqueous, soluble oligosaccharide substrates [28]. Considering that the recombinant sialidases used in this study differed in their modular domain architecture (BbSia2α containing three lectin-like β-sandwich fold domains – one N-terminal to and two C-terminal to the sialidase domain (AlphaFoldDB ID: A0A1Y1C8G4), CpNanI expressed as a single sialidase domain without its native co-occurring N-terminal CBM40 domain (PDB ID: 5TSP; AlphaFoldDB ID: A0A0H2YQR1), and HpNanH containing a central alpha-helical bundle domain and a C-terminal aquaporin-like domain in addition to the N-terminal sialidase domain (AlphaFoldDB ID: I7AJQ9)), we cannot rule out the possibility that these co-occurring domains might have influenced substrate preference by specifically enhancing binding to a sialylated oligosaccharide with a specific glycosidic linkage.

Our study indicates that BbSia2α and CpNanI, and probably many more sialidases can hydrolyze oligosaccharides such as LSTb that contain the Siaα2-6GlcNAc glycosidic linkage. The Siaα2-6GlcNAc linkage is an interesting sialylation of “internal” GlcNAc residues [29], that has been reported not only in the HMOs, DSLNT and LSTb, but also in the N-glycans of bovine prothrombin [30], fetuin [31], alpha-A1-acid from different animals [32,33] and glycoproteins found in Artiodactyla sera [34], and in glycolipids, such as a ganglioside (ceramide derivative of LSTb) in human meconium [35], and disialyl Lewis A containing glycolipids which are preferentially expressed in normal colonic epithelial cells compared to malignant cells [36]. Sialyltransferases capable of catalyzing this linkage have been reported in regenerating rat liver and other tissues [37,38].

The Siaα2-6GlcNAc glycosidic linkage is especially of interest as it is present in the HMO, DSLNT (Fig. 1a), whose abundance in human milk is inversely associated with the occurrence of necrotizing enterocolitis (NEC) in neonates [12,[39], [40], [41], [42]], and which is reported to protect the intestinal epithelium of infants against inflammatory bowel disease (IBD) [43]. Commensal bacteria that secrete sialidases capable of hydrolyzing the Siaα2-6GlcNAc linkage are, therefore, likely to colonize better and contribute to gut health. Future studies aimed at screening for the ability to hydrolyze HMOs containing the Siaα2-6GlcNAc linkage might therefore be useful in identifying probiotics for NEC, IBD, and other intestinal diseases. Further, considering the reported use of sialidases for the synthesis of sialylated HMOs by *trans*-sialylation reactions, and considering that some sialidases show similar glycosidic linkage preferences in the hydrolysis and *trans*-sialylation reactions [[44], [45], [46]], it will be interesting to assess in future studies if the potential therapeutic HMO, DSLNT, can be synthesized using BbSia2α or CpNanI or other sialidases capable of hydrolyzing the Siaα2-6GlcNAc linkage in LSTb.

Finally, characterizing and making available the substrate preference of sialidases concerning the sub-terminal reducing-end saccharide moiety in addition to the glycosidic linkage α2-3 or α2-6, especially for frequently used sialidases, will enable a better selection of enzymes for different applications.

## 5 Data availability

Image data supporting the findings of this study are available within this article. Other raw data are available with the corresponding author and will be shared upon reasonable request.

## Funding

This work was supported by the Council of Scientific and Industrial Research, Government of India (CSIR-IMTECH Research Council approved project OLP0178 to TNCR). ML acknowledges the Council of Scientific and Industrial Research, Government of India for her fellowship.

## CRediT authorship contribution statement

**Madhu Lata:** Writing – review & editing, Writing – original draft, Visualization, Validation, Methodology, Investigation, Formal analysis, Data curation. **T.N.C. Ramya:** Writing – review & editing, Supervision, Funding acquisition, Formal analysis, Conceptualization.

## Declaration of competing interest

The authors declare that they have no known competing financial interests or personal relationships that could have appeared to influence the work reported in this paper.
