# Supplementary material for: A comparative study of the substrate preference of the sialidases, CpNanI, HpNanH, and BbSia2 towards 2-Aminobenzamide-labeled 3′-Sialyllactose, 6′-Sialyllactose, and Sialyllacto-N-tetraose-b
Source: Biochem Biophys Rep. 2024 Jul 19;39:101791. doi: 10.1016/j.bbrep.2024.101791 (PMC11326918; doi:10.1016/j.bbrep.2024.101791)
Supplement: Multimedia component 1 [file mmc1.pdf]

Supplementary Information

A comparative study of the substrate preference of the sialidases, *Cp*NanI, *Hp*NanH, and *Bb*Sia2 towards 2-Aminobenzamide-labeled 3′-Sialylactose, 6′-Sialyllactose, and Sialyllacto-N-tetraose-b

Madhu Lata[1,2] and T.N.C. Ramya [*,1,2]

[1]CSIR- Institute of Microbial Technology, Sector 39-A, Chandigarh 160036, INDIA

[2] Academy of Scientific & Innovative Research (AcSIR), Ghaziabad, Uttar Pradesh 201002, India.

*Correspondence to be addressed to T.N.C. Ramya, CSIR- Institute of Microbial Technology, Sector 39-A, Chandigarh 160036, INDIA. Tel: 91-172-2880243; E-mail: ramya@imtech.res.in

Running Title: Substrate preference of sialidases

This supplementary information file contains two supplementary figures, Fig. S1 and Fig. S2.

Fig. S1

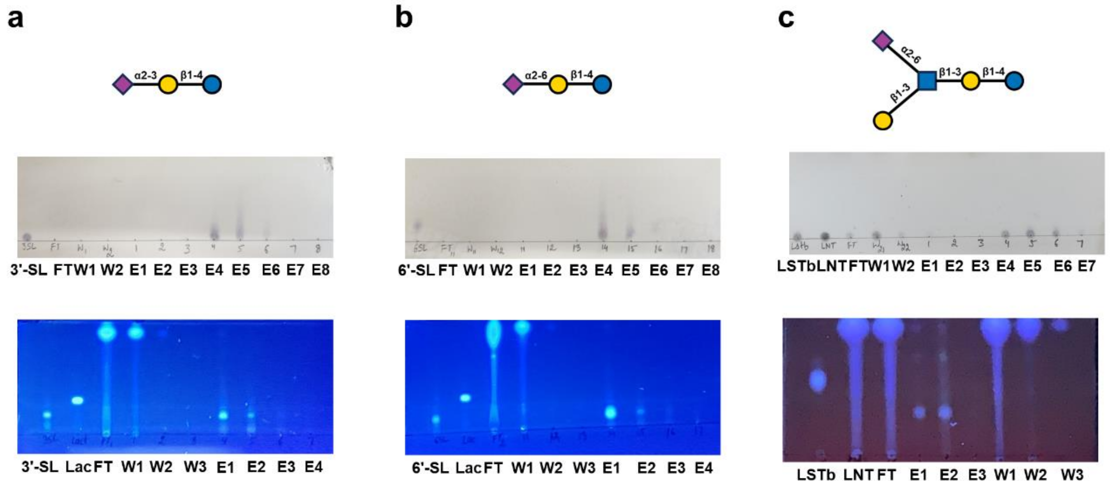

Purification and labeling of oligosaccharides. Upper panel: DPA-stained TLC plates showing the various fractions collected during the purification of 3′-SL (A), 6′-SL (B) and LSTb (C) from unsialylated contaminants using Dowex AG1X-8 resin. FT: flowthrough; W1, W2: washes with water; E1-E2: eluates with 0.01 M formic acid; E3-E4: eluates with 1 M formic acid; E5-E6: eluates with 4 M formic acid; E7-E8: eluates with 4 M formic acid and 0.04 M ammonium formate. Lower panel: UV-fluorescence of TLC plates showing the various fractions collected during the purification of 2-AB labeled- 3′-SL (A), 6′-SL (B), and LSTb (C) using HILIC SPE. FT: flowthrough; W1-W3: washes with 96% acetonitrile; E1-E4: eluates with water. The TLC solvent used was Butanol: Acetic Acid : Water:: 2:1:1.

Fig. S2

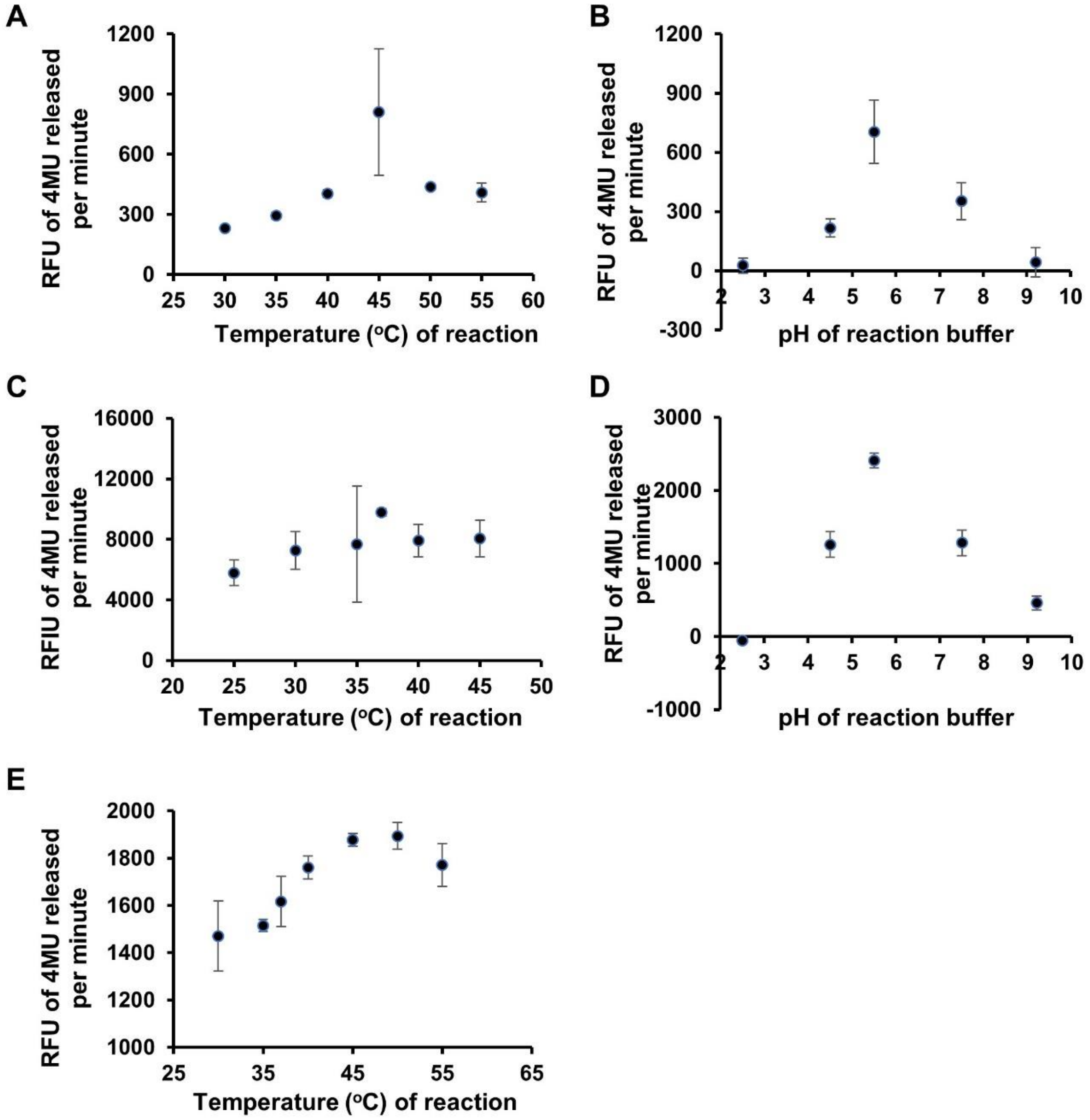

Optimization of temperature and pH conditions for sialidase assays. (A, B) Sialidase activity of CpNanI at different temperatures and pH. (C, D) Sialidase activity of HpNanH at different temperatures and pH. (E) Sialidase activity of BbSia2α at different temperatures. The optimal temperature and pH for the sialidase reaction were determined using 5 μM (BbSia2α) or 10 μM (CpNanI and HpNanH) 4-MU-Neu5Ac and 0.01 nM (CpNanI), or 0.01 μM (HpNanH) or 0.25 nM (BbSia2α) protein. For optimizing temperature, 50 mM sodium acetate, pH 5.5 was used. For optimizing pH, 45 °C (CpNanI) and 37 °C (HpNanH) were used. The following

buffers were used for the different pH conditions - 100 mM Glycine-HCl (pH 2.5), 50 mM sodium acetate (pH 4.5 or pH 5.5), 20 mM Tris(hydroxymethyl)aminomethane in 150 mM sodium chloride (pH 7.5), and 100 mM Glycine-NaOH (pH 9.2).
